# Fear of Falling: Exploring Associated Factors among Elderly Residents in the Rural Communities of Vietnam

**DOI:** 10.3390/ijerph21060691

**Published:** 2024-05-28

**Authors:** Luc Viet Tran, Thanh Xuan Nguyen, Thu Thi Hoai Nguyen, Huong Thi Thu Nguyen, Tam Ngoc Nguyen, Thao Thi Phuong Nguyen, Huong Thi Thanh Nguyen, Thang Pham, Anh Trung Nguyen, Huyen Thi Thanh Vu

**Affiliations:** 1 Neurology Department, Hanoi Medical University, Hanoi 100000, Vietnam; xuanthanhbmlk@hmu.edu.vn (T.X.N.); nththu.bvlk@gmail.com (T.T.H.N.); thuhuonglk@hmu.edu.vn (H.T.T.N.); ngoctam@hmu.edu.vn (T.N.N.); phamthang@hmu.edu.vn (T.P.); trunganhvlk@gmail.com (A.T.N.); vuthanhhuyen11@hmu.edu.vn (H.T.T.V.); 2 Scientific Research Department, National Geriatric Hospital, Hanoi 100000, Vietnam; 3 Geriatrics Department, Hanoi Medical University, Hanoi 100000, Vietnam; 4 Institute for Preventive Medicine and Public Health, Hanoi Medical University, Hanoi 100000, Vietnam; dr.nguyenthiphuongthao.hmu@gmail.com; 5 Academy of Medical Sciences, Ho Chi Minh 700000, Vietnam; 6 Dinh Tien Hoang Institute of Medicine, Hanoi 100000, Vietnam; ntthanhhuong@hmu.edu.vn; 7 Physiology Department, Hanoi Medical University, Hanoi 100000, Vietnam

**Keywords:** fear of falling, older people in community, Vietnam, risk of fall

## Abstract

This research aimed to explore factors associated with the fear of falling (FOF) among community-dwelling older adults in Vietnam. A cross-sectional study was conducted in five communes in Soc Son, Hanoi, Vietnam, from March to June 2017. We recruited a total of 487 participants, which provided sufficient data for analysis. The outcome variable was fear of falling. Several covariates, including demographics, medical history, general health status, geriatric syndromes, eye diseases, assessment of fall risk environment, timed up-and-go test, and number of standing up in 30 s, were collected. A multivariable logistic regression model was performed to determine predictors associated with FOF. The results showed that 54.6% of the participants had FOF. Furthermore, the logistic multivariable regression model revealed several factors associated with FOF among participants in the research sites, including polypharmacy status (OR: 1.79; 95%CI 1.07–2.99), higher scores in quality of life according to the EQ-5D-5L index (OR:6.27; 95%CI: 2.77–14.17), and having fallen during the past 12 months (OR:4.4; 95%CI: 2.39–8.11). These findings contribute to a comprehensive understanding of the intricate relationship between FOF and several associated factors, notably polypharmacy status, quality of life, and having a fall during the past 12 months.

## 1. Introduction

Falls among the elderly comprise a significant public health concern, precipitating not only health-related complications but also imposing a substantial societal burden. Such occurrences can lead to disabilities, hospitalizations, increasing healthcare expenditures, and mortality [1]. It can lead to increased levels of functional dependency, fear of falling, and poorer quality of life [2,3,4]. It is estimated that 35–45% of people over 65 years old report falling each year in the community and this proportion rises to 50% among those over 80 years old [2,5]. In Vietnam, a previous study indicated that the prevalence of falls in older people was 23.7%, of which the rate of recurrent falls was 5.8% [6].

Fear of falling (FOF) is a syndrome defined as “an ongoing concern about falling that ultimately leads to avoidance of the performance of daily activities” [4]. FOF is more prevalent in old age and may lead to a loss of self-confidence and reduced physical and social activities, resulting in increased dependency [7,8]. In addition, FOF may lead to a decreased balance [9], mental impairment [10,11], falls and recurrent falls, and institutionalization [7,12]. About 3–85% of older adults have FOF [13]. The prevalence of FOF in the elderly ranges between 12% and 65% in community-dwelling older adults and 30.0% and 62.8% in the elderly admitted to the hospital [7,14]. Older adults with a history of falls are twice as likely to be afraid of falling than those without a history of falls [15,16]. Meanwhile, older adults without a previous history of falls still expressed some FOF [7]. Therefore, FOF is considered both a major cause and consequence of falls.

FOF and its associated factors are crucial considerations in interventions aimed at mitigating the impact of falls and their consequences [17]. Research in developed countries has extensively explored the significance of FOF and its repercussions as key determinants influencing health outcomes [7,18]. Moreover, comorbidity, the coexistence of multiple chronic conditions within an individual, is increasingly recognized as a significant challenge in healthcare, particularly among aging populations. This phenomenon exacerbates the complexity of managing health outcomes and poses substantial burdens on both individuals and healthcare systems [19,20]. Research indicates that individuals with comorbidity are at higher risk of experiencing falls [20,21]. The interplay between comorbidity and falls underscores the need for comprehensive assessment and tailored interventions to address the multifaceted health needs of older adults.

Despite the growing body of knowledge in developed nations, there is a notable gap in understanding the relationship between FOF and factors related to fear of falling among the elderly in developing countries like Vietnam. Limited studies conducted in Vietnamese community-dwelling older adults have reported FOF prevalence rates ranging from 40.8% to 64% [16,22]. However, the specific impact of FOF on various aspects associated with fear of falling in older individuals remains unclear. The current state of knowledge is insufficient for optimizing health interventions designed to enhance factors related to the fear of falling among older people. Hence, this research was undertaken to explore factors associated with the fear of falling among community-dwelling older adults in Vietnam.

## 2. Materials and Methods

### 2.1. Design, Setting, and Sampling 

A cross-sectional study was conducted in five communes, in Soc Son district, Hanoi, Vietnam, from March to June 2017. We used the simple random sampling method to recruit participants from an existing list of individuals aged 60 and over.

The sample size was determined utilizing a formula based on a single population proportion: *n* = Z^2^ _1-α/2_ × [p*(1 − p)/d^2^], where *n* represents the minimum sample size, Z_1-α/2_ = 1.96 (with α = 0.05 and 95% confidence interval), and p denotes the proportion estimated from a prior study in Central Vietnam (p = 0.41) [16]. Therefore, our study required a sample size of at least 371 participants. To account for potential incomplete responses or dropouts, the sample size was increased by 10%. Ultimately, we recruited a total of 487 participants, providing sufficient data for analysis. Information was collected via in-person interviews and measurements conducted with the patients, utilizing a pre-defined data collection form.

### 2.2. Measure and Instruments

#### 2.2.1. Outcome Variables

##### Fear of Falls

Fear of falling was assessed by using the question “Do you have fear of falling?” with a “Yes/No” answer. This approach has been used in previous studies. It is simple and feasible to collect the responses from the study population in the community setting.

#### 2.2.2. Covariables

##### Demographic/General Characteristics

The characteristics such as age, gender, marital status, living status, caregiver, smoking status, and alcohol consumption were noted.

##### Medical History and General Health Status

Medical history included instances of urge incontinence, eye diseases, and health examinations in the last 12 months. Additionally, the number of medications was recorded using medical documents and interviews with the participants, with polypharmacy defined as the routine use of five or more medications. The Mini-cog test, comprising a three-word recall and Clock test, was utilized to evaluate cognitive impairment. Frailty was assessed using the Fried criteria, with three or more criteria indicating the presence of frailty syndrome. The assessment of nutritional status was conducted utilizing the Mini Nutritional Assessment Short Form (MNA-SF) instrument, with scores ranging from 0 to 14 points and categorized as follows: ≤7 points for malnourished status, 8–11 points for risk of malnourishment, and ≥12 points for normal status [23].

##### Health-Related Quality of Life

The Health-related Quality of Life (HrQoL) was evaluated using the Vietnamese version of the EQ-5D-5L. This scale consists of five dimensions—mobility, self-care, usual activities, pain/discomfort, and anxiety/depression—each categorized into five levels of functional classification. The EQ-5D-5L index of the study population was determined using Vietnamese classification, with values ranging between −0.512 (representing the worst possible HrQoL) and 1 (representing the best possible HrQoL); a value < 0 indicates a health state valued worse.

##### Assessment of Fall Risk Environment

History of fall in the last twelve months (yes/no) was recruited. The surrounding living environment was assessed: using walking aids, getting on/off the bed, going upstairs, bathroom mobility barriers, improper furniture design, narrow indoor path, and indoor light.

Participants were interviewed according to eight function domains outlined by the Instrumental Activities of Daily Living (IADL). This tool primarily assesses the current functional activity levels of patients. Men are evaluated excluding food preparation, housekeeping, or laundry, whereas women are rated across all eight functional areas. Each participant is scored based on their highest performance level within each domain [17]. The resulting summary score ranges from 0 (indicating poor function and dependency) to 8 (representing excellent function and independence).

The timed up-and-go test (TUG) was employed to evaluate balance, walking capability, and functional mobility among elderly individuals. Participants recorded the time (seconds) it took to complete the test.

A 30 s sit-to-stand test was used to assess leg strength and endurance. The number of times the participants stood in 30 s was recorded.

### 2.3. Statistical Analysis

Data analysis was conducted using STATA version 16 (StataCorp. LP, College Station, TX, USA). Continuous variables were expressed as mean (±standard deviation), while categorical variables were presented as frequency and percentage. Chi-square tests were employed for comparing categorical variables between participants with and without FOF, while the Whitney–Wilcoxon test was used for continuous variables. Statistical significance was set at *p* values < 0.05.

To construct a multivariable model, all potential covariates associated with FOF categorization were thoroughly examined and assessed. Univariate logistic regression models were employed to establish the unadjusted odds ratios between FOF and the predictors. The multivariable logistic regression model included all variables found to be significant in the unadjusted models. Logistic regression analysis was conducted to identify predictors linked with FOF (coded as Yes = 1/No = 0). Stepwise forward methods were employed to streamline and refine these models, with a *p*-value > 0.2 serving as the threshold for excluding variables. Statistical significance was determined by a *p*-value < 0.05. Several good fit models were used to estimate the final model including Pseudo R2, Akaike Information Criterion (AIC), Bayesian Information Criterion (BIC), Hosmer–Lemeshow, etc. The confusion matrix assesses a classification model’s performance for the fear of falling outcomes [Supplementary Table S1]. The Under the Receiver Operating Characteristic Curve (AUC) gauges the model’s ability to distinguish between positive and negative classes based on the threshold for fear of falling [Supplementary Figure S1].

## 3. Results

Among 487 participants, the mean age was 70.9 years (SD ± 8.3). Most of the patients were females (70.2%), married (64.5%), lived with family (94.7%), and were cared for by their spouse/children (83.6%). Most participants reported not smoking (90.3%) and not drinking alcohol (79.1%). A statistically significant difference between those with and without FOF was found in the variables of gender, marital status, and living arrangements (spouse/children, grandchildren/great-grandson, nursing home, domestic servant) with *p*-values ≤ 0.05 (Table 1).

Table 2 demonstrates medical history, general health status, and quality of life according to FOF status. The majority of participants experienced FOF, accounting for 54.6%. Respondents reported having periodic health examinations (69.8%), taking four or fewer types of medicine (74.7%), experiencing urge incontinence (27.1%), and having eye diseases (32.2%). Furthermore, most participants were categorized into cognitive impairment groups according to the Mini-cog scale (54.1%), at risk of malnutrition (56.3%), and frail (77.2%). The mean EQ-5D-5L score was −0.1 (SD = ±0.3). The differences in polypharmacy status, nutritional assessment, frailty status (Fried frailty criteria), and EQ-5D-5L index between the two groups of “with or without FOF” were statistically significant (*p* < 0.05).

Table 3 shows the assessment of fall risk among participants. The percentage of people who had fallen during the past 12 months accounted for 21.1%. Participants reported being able to get on and off the bed easily and safely (74.9%), go upstairs easily (85.2%), encounter bathroom mobility barriers (53.0%), and face indoor light mobility barriers (96.7%). People with FOF were also found to be dependent on daily living (16.9%). The mean timed up-and-go score and number of stand-ups in 30 s was, respectively, 11.6 (SD ± 3.4) and 4.6 (SD ± 1.4). Having fallen during the past 12 months, risk of falling, ease of getting on and off the bed safely, improper furniture design, dependence on daily living, longer timed up-and-go duration, and a higher number of stand-ups in 30 s were significantly associated with fear of falling (*p*-value ≤ 0.05).

In the multivariable logistic regression model (Table 4), the factors associated with having FOF include having polypharmacy status (OR = 1.57; 95%CI: 0.96–2.58); higher scores in quality of life according to EQ-5D-5L index (OR = 5.51; 95%CI: 2.35–12.91); living alone without caregiver (OR = 4.31; 95%CI: 1.08–17.21); and having a fall during the past 12 months (OR = 5.27; 95%CI: 2.72–10.23).

## 4. Discussion

This study was conducted to highlight the prevalence of FOF and several related factors among the elderly in a district of Hanoi, Vietnam. The results showed that 54.6% of the participants had FOF. Furthermore, the logistic multivariable regression model revealed several factors associated with the FOF among the elderly in the research sites, which include having polypharmacy status, higher scores in quality of life according to the EQ-5D-5L index, and having a fall during the past 12 months.

This study reveals that 54.6% of participants in the rural districts of Vietnam experience FOF, a noteworthy finding that prompts insightful comparisons with global and regional studies. In developed countries, FOF prevalence ranges from 3% to 85%, placing the observed rate within this spectrum [24,25]. It is crucial to explore potential regional variations within Vietnam and compare urban and rural disparities. The study’s identified risk factors, including living alone without a caregiver, polypharmacy, higher quality of life scores, and a history of falls, should be contextualized through comparisons with findings from similar studies in Vietnam and neighboring nations. However, research on the relationship between FOF among the elderly in developing countries, such as Vietnam, is scarce. Currently, only a few studies have been conducted on Vietnamese community-dwelling older adults, revealing FOF prevalence rates of 40.8% and 64%. This paucity of research underscores the need for a more comprehensive understanding of how FOF and its influences within the specific socio-cultural context of Vietnam [16,22].

A more comprehensive understanding of the factors influencing FOF and inform the development of effective interventions tailored to the specific needs of elderly individuals in this context. Firstly, the association between polypharmacy and FOF was observed, with individuals having polypharmacy status showing a higher likelihood of experiencing FOF (OR = 1.55; 95%CI: 0.95–2.52). The association between polypharmacy and comorbidity is evident, as individuals with multiple health conditions often require multiple medications. Over recent years, numerous studies have explored the relationship between comorbidity and falls, with findings consistently indicating a positive relationship between the two factors [26,27,28]. Additional viewpoints have indicated that polypharmacy exacerbates the likelihood of falls, potentially attributed to factors like orthostatic hypotension and cognitive confusion [29,30]. Thus, this finding emphasizes the need for a comprehensive medication management approach in the elderly population to address the potential side effects and interactions that may compromise their overall well-being [31]. Secondly, a history of falls during the past 12 months was a significant predictor of FOF (OR = 5.39; 95%CI: 2.87–10.12). This aligns with the existing literature, emphasizing the bidirectional relationship between FOF and actual falls. The experience of a fall can instill a persistent fear, hindering an individual’s confidence in their ability to perform daily activities without the risk of subsequent falls [32].

Surprisingly, higher scores in the quality of life according to the EQ-5D-5L index were associated with an increased likelihood of FOF (OR = 5.61; 95%CI: 2.42–13.01). One possible interpretation is that individuals with elevated quality-of-life scores may possess heightened awareness of their health status, making them more attuned to the potential consequences of a fall. This increased awareness could contribute to a heightened FOF, as individuals with a more positive perception of their well-being may be particularly concerned about any potential threat to their health and independence [17,33]. This finding prompts further exploration into the underlying mechanisms linking FOF and quality of life. Future research may delve into the psychosocial aspects of FOF, examining how it intersects with self-perception, mental well-being, and the broader concept of health-related quality of life. Such insights are crucial for tailoring interventions that not only address the physical aspects of falls but also consider the intricate interplay between psychological factors and perceived quality of life among the elderly in this specific cultural context.

This study has several limitations that warrant consideration. The cross-sectional design impedes the establishment of causal relationships, highlighting the need for future longitudinal research to explore the dynamic nature of FOF over time. Reliance on self-reported measures, such as FOF and quality of life, introduces potential biases related to recall and subjective interpretation. Additionally, this study’s focus on a specific rural district in Vietnam may limit the generalizability of findings to other contexts within the country or different cultural settings.

## 5. Conclusions

In conclusion, the present results highlight the multifaceted nature of FOF among the elderly in a rural area of Vietnam. Interventions aimed at reducing FOF should encompass social support mechanisms, targeted medication management strategies, and comprehensive fall prevention programs. Further research is warranted to elucidate the complex interplay between subjective perceptions of quality of life and FOF and to determine whether these associations are consistently observed in different populations facilitating the development of tailored interventions for this vulnerable population.

## Figures and Tables

**Table 1:** General characteristics of participants (*n* = 487).

Characteristics	No Fear of Falling		Fear of Falling		Total		*p*-Value
	*n*	%	*n*	%	*n*	%	
**Total**	221	45.4	266	54.6	487	100.0	
**Gender**							
Male	79	35.7	66	24.8	145	29.8	0.009
Female	142	64.3	200	75.2	342	70.2	
**Marital status**							
Single/widowed/divorced	61	27.6	112	42.1	173	35.5	0.001
Having Spouse	160	72.4	154	57.9	314	64.5	
**Living with**							
Family	216	97.7	245	92.1	461	94.7	0.011
Brothers/sisters/relatives	2	0.9	1	0.4	3	0.6	
Alone with caregiver	0	0.0	5	1.9	5	1.0	
Alone without caregiver	3	1.4	15	5.6	18	3.7	
**Caregiver**							
Spouse/children	189	85.5	218	82.0	407	83.6	0.192
Grandchildren/great-grandson	10	4.5	24	9.0	34	7.0	
Nursing home	0	0.0	1	0.4	1	0.2	
Domestic servant	22	10.0	23	8.6	45	9.2	
**Smoking status**							
No	197	89.1	243	91.4	440	90.3	0.528
Yes but gave up	11	5.0	8	3.0	19	3.9	
Yes	13	5.9	15	5.6	28	5.7	
**Alcohol use state**							
No	168	76.0	217	81.6	385	79.1	0.137
Yes but gave up	5	2.3	9	3.4	14	2.9	
Yes	48	21.7	40	15.0	88	18.1	
	Mean	SD	Mean	SD	Mean	SD	*p*-value
**Age**	70.4	7.7	71.3	8.8	70.9	8.3	0.474

SD: standard deviation.

**Table 2:** Medical history, general health status, and quality of life according to fear of falling status (*n* = 487).

Characteristics	No Fear of Falling		Fear of Falling		Total		*p*-Value
	*n*	%	*n*	%	*n*	%	
**Total**	221	45.4	266	54.6	487	100.0	
**Health examination in the last 12 months**							
Yes	148	67.0	192	72.2	340	69.8	0.212
No	73	33.0	74	27.8	147	30.2	
**Polypharmacy status**							
Take four types of medicine or less	168	76.0	196	73.7	364	74.7	0.555
Take more than five types of medicine	53	24.0	170	26.3	123	25.3	
**Urge incontinence**							
No	166	75.1	189	71.1	355	72.9	0.316
Yes	55	24.9	77	28.9	132	27.1	
**Eye diseases**							
No	122	55.2	136	51.1	258	53.0	0.253
Yes	63	28.5	94	35.3	157	32.2	
Unknown	36	16.3	36	13.5	72	14.8	
**Cognitive impairment status (Mini-cog scale)**							
Lower likelihood of dementia	95	43.0	127	47.7	222	45.6	0.294
Cognitive impairment	126	57.0	139	52.3	265	54.4	
**Nutritional assessment (MNA-SF)**							
Malnutrition	19	8.6	58	21.8	77	15.8	0.000
Risk of malnutrition	124	56.1	150	56.4	274	56.3	
Good nutrition	78	35.3	58	21.8	136	27.9	
**Frailty status (Fried frailty criteria)**							
Non-frail	30	15.7	72	28.0	102	22.8	0.002
Frail	161	84.3	185	72.0	346	77.2	
	Mean	SD	Mean	SD	Mean	SD	*p*-value
**EQ-5D-5L index (score)**	−0.3	0.3	−0.1	0.3	−0.1	0.3	0.000

SD: Standard Deviation.

**Table 3:** Assessment of fall risk among participants (*n* = 487).

Characteristics	No Fear of Falling		Fear of Falling		Total		*p*-Value
	*n*	%	*n*	%	*n*	%	
**Total**	221	45.4	266	54.6	487	100.0	
**Having a fall during the past 12 months**							
No	204	92.3	180	67.7	384	78.9	0.000
Yes	17	7.7	86	32.3	103	21.1	
**Get on and off the bed easily and safely**							
No	33	14.9	89	33.5	122	25.1	0.000
Yes	188	85.1	177	66.5	365	74.9	
**Going upstairs easily**							
No	29	13.1	43	16.2	72	14.8	0.346
Yes	192	86.9	223	83.8	415	85.2	
**Bathroom mobility barriers**							
No	101	45.7	128	48.1	229	47.0	0.594
Yes	120	54.3	138	51.9	258	53.0	
**Improper furniture design**							
No	189	85.5	208	78.2	397	81.5	0.038
Yes	32	14.5	58	21.8	90	18.5	
**Narrow indoor path**							
No	186	84.2	207	77.8	393	80.7	0.077
Yes	35	15.8	59	22.2	94	19.3	
**Indoor light mobility barriers**							
No	5	2.3	11	4.1	16	3.3	0.248
Yes	216	97.7	255	95.9	471	96.7	
**Dependence on daily living (IADL)**							
Independence	208	94.1	221	83.1	429	88.1	0.000
Dependence	13	5.9	45	16.9	58	11.9	
	Mean	SD	Mean	SD	Mean	SD	
**Timed up-and-go test (second)**	10.9	2.6	12.2	3.9	11.6	3.4	0.000
**Number of stand-ups** **in 30 s**	4.4	1.0	4.8	1.6	4.6	1.4	0.000

SD: Standard Deviation.

**Table 4:** Using the logistic regression model to identify the related factors of fear of falling among participants.

Variables	Fear of Falling (vs. No)					
	Univariate Logistic Regression Model			Multivariable Logistic Regression Model		
	OR	95% CI	*p*-Value	OR	95% CI	*p*-Value
**Gender** (ref: Male)						
Female	1.69	(1.14–2.49)	0.009	1.63	(0.79–3.36)	0.186
**Living with** (ref: Family)						
Brothers/sisters/relatives	0.44	(0.04–4.90)	0.505	0.09	(0.01–2.28)	0.153
Alone with caregiver	1			1		
Alone without caregiver	4.40	(1.26–15.43)	0.020	4.31	(1.08–17.21)	0.039
**Alcohol use state** (ref: No)						
Yes but gave up	1.39	(0.46–4.24)	0.558	2.76	(0.68–11.20)	0.155
Yes	0.65	(0.41–1.03)	0.065	1.07	(0.48–2.40)	0.867
**Alcohol use state** (ref: No)						
Yes but gave up	1.39	(0.45–4.23)	0.558	2.76	(0.68–11.20)	0.155
Yes	0.64	(0.40–1.03)	0.065	1.07	(0.48–2.40)	0.867
**Age** (ref: Unit)	1.01	(0.99–1.04)	0.233	0.99	(0.96–1.03)	0.696
**Periodic health examination** (per: Yes)						
No	0.213	(0.53–1.15)	0.213	0.85	(0.53–1.37)	0.507
**Polypharmacy status** (ref: Take four types of medicine or less)						
Take more than five types of medicine	1.13	(0.75–1.71)	0.555	1.57	(0.96–2.58)	0.074
**Urge incontinence** (ref: No)						
Yes	1.22	(0.82–1.84)	0.316	0.91	(0.56–1.50)	0.738
**Eye diseases** (ref: No)						
Yes	1.33	(0.90–2.00)	0.155	1.19	(0.72–1.94)	0.487
Unknown	0.90	(0.53–1.51)	0.684	0.57	(0.29–1.10)	0.094
**Cognitive impairment status (Mini-cog scale)** (ref: Lower likelihood of dementia)						
Cognitive impairment	0.83	(0.58–1.18)	0.294	1.57	(0.98–2.51)	0.059
**Nutritional assessment** (ref: Malnutrition)						
Risk of malnutrition	0.40	(0.22–0.70)	0.001	0.45	(0.22–0.89)	0.022
Good nutrition	0.24	(0.13–0.45)	0.000	0.31	(0.15–0.67)	0.003
**Having a fallen during past 12 months** (ref: No)						
Yes	5.73	(3.28–10.01)	0.000	5.27	(2.72–10.23)	0.000
**Risk of fall according to Morse Fall Scale** (ref: Low risk)						
Medium risk	2.01	(1.19–3.41)	0.009	1.07	(0.54–2.23)	0.840
High risk	4.81	(2.35–9.84)	0.000	2.02	(0.81–5.04)	0.130
**Get on and off the bed easily and safely** (ref: No)						
Yes	0.35	(0.22–0.55)	0.000	0.35	(0.21–0.59)	0.000
**Narrow indoor path** (ref: No)						
Yes	1.52	(0.95–2.41)	0.079	1.43	(0.33–6.28)	0.631
**Indoor light mobility barriers** (ref: No)						
Yes	0.54	(0.18–1.57)	0.255	1.08	(0.95–1.21)	0.236
**Timed up-and-go test** (ref: Per score)	1.13	(1.06–1.21)	0.000	0.96	(0.73–1.26)	0.774
**EQ-5D-5L index** (ref: Per score)	12.26	(6.10–24.67)	0.000	5.51	(2.35–12.91)	0.000
**Fit models of multivariable logistic regression model (adjusted model)**				Value		Fit value
**Pseudo R2**				0.21		0.2–0.4
**Akaike Information Criterion (AIC)**				568.99		
**Bayesian Information Criterion (BIC)**				669.263		
***p*-value (Hosmer–Lemeshow)**				0.019		<0.05

OR: Odds Ratio; CI: Confidence Interval.

## Data Availability

The data presented in this study are available on request from the corresponding author due to privacy.

## Supplementary Materials

The following supporting information can be downloaded at: https://www.mdpi.com/article/10.3390/ijerph21060691/s1, Figure S1: The Under the Receiver Operating Characteristic Curve (AUC) measures the ability of the model to distinguish between positive and negative classes across the threshold value of the Fear of Fall outcome.; Table S1: The confusion matrix to evaluate the performance of a classification.
