# Supplementary material for: Fear of Falling: Exploring Associated Factors among Elderly Residents in the Rural Communities of Vietnam
Source: Int J Environ Res Public Health. 2024 May 28;21(6):691. doi: 10.3390/ijerph21060691 (PMC11203546; doi:10.3390/ijerph21060691)
Supplement: Supplementary file 1 [file ijerph-21-00691-s001.zip › ijerph-2994763-supplementary.pdf]

SUPPLEMENTARY TABLE S1: The confusion matrix to evaluate the performance of a classification model of fear of falling outcome.

**Fear of falling (FOF)**		**Predicted**		
		**Negative** (No FOF)	**Positive** (FOF)	**Total**
**Actual**	**Negative** (No FOF)	185	57	242
	**Positive** (FOF	76	164	240
	**Total**	261	221	482
**CLASSIFIED**			**VALUE**	
Sensitivity	**Predicted** (+| No FOF)		70.88%	
Specificity	**Predicted** (-| FOF)		74.21%	
Positive predictive value	**Predicted (**No FOF| **+)**		76.45%	
Negative predictive value	**Predicted** (FOF | -)		68.33%	
False + rate for true FOF	**Predicted** (+| FOF)		25.79%	
False - rate for true No FOF	**Predicted** (-| No FOF)		29.12%	
False + rate for classified +	**Predicted** (FOF | +)		23.55%	
False - rate for classified -	**Predicted** (No FOF | -)		31.67%	
**Correctly classified**			**72.41%**	

SUPPLEMENTARY FIGURE S1: The Under the Receiver Operating Characteristic Curve (AUC) measures the ability of the model to distinguish between positive and negative classes across the threshold value of the Fear of Fall outcome.

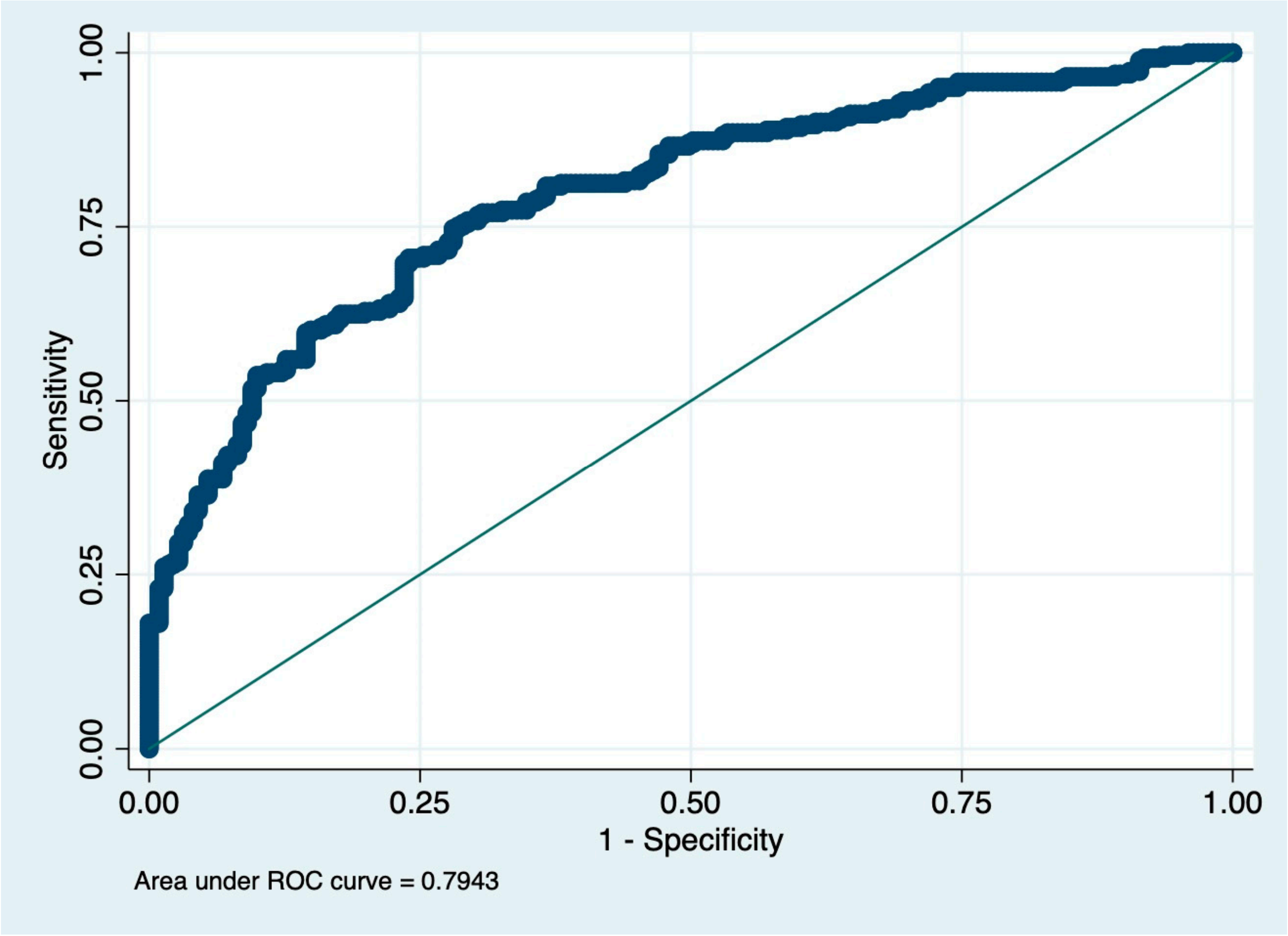
1.00
0.75
0.50
0.25
0.00
Sensitivity
0.00
0.25
0.50
0.75
1.00
1 - Specificity
Area under ROC curve = 0.7943
